# Long Noncoding RNA LINC00578 Inhibits Ferroptosis in Pancreatic Cancer via Regulating SLC7A11 Ubiquitination

**DOI:** 10.1155/2023/1744102

**Published:** 2023-02-14

**Authors:** Haoran Li, Yijun Wei, Jie Wang, Jun Yao, Chen Zhang, Chengqing Yu, Yuchen Tang, Dongming Zhu, Jian Yang, Jian Zhou

**Affiliations:** ^1^Department of General Surgery, The First Affiliated Hospital of Soochow University, Suzhou, Jiangsu 215006, China; ^2^Department of General Surgery, The Dushu Lake Hospital Affiliated to Soochow University, Suzhou, Jiangsu 215006, China

## Abstract

**Background:**

Pancreatic cancer is a highly aggressive malignancy worldwide with rapid development and an exceedingly poor prognosis. lncRNAs play crucial roles in regulating the biological behaviors of tumor cells. In this study, we discovered that LINC00578 acted as a regulator of ferroptosis in pancreatic cancer.

**Methods:**

A series of loss- and gain-of-function experiments in vitro and in vivo were performed to explore the oncogenic role of LINC00578 in pancreatic cancer development and progression. Label-free proteomic analysis was performed to select LINC00578-related differentially expressed proteins. Pull-down and RNA immunoprecipitation assays were carried out to determine and validate the binding protein of LINC00578. Coimmunoprecipitation assays were used to investigate the association of LINC00578 with SLC7A11 in ubiquitination and to confirm the interaction between ubiquitin-conjugating enzyme E2 K (UBE2K) and SLC7A11. An immunohistochemical assay was used to confirm the correlation between LINC00578 and SLC7A11 in the clinic.

**Results:**

LINC00578 positively regulated cell proliferation and invasion in vitro and tumorigenesis in vivo in pancreatic cancer. LINC00578 can obviously inhibit ferroptosis events, including cell proliferation, reactive oxygen species (ROS) generation, and mitochondrial membrane potential (MMP) depolarization. In addition, the LINC00578-induced inhibitory effect on ferroptosis events was rescued by SLC7A11 knockdown. Mechanistically, LINC00578 directly binds UBE2K to decrease the ubiquitination of SLC7A11, thus accelerating SLC7A11 expression. In the clinic, LINC00578 is closely associated with clinicopathologic factors and poor prognosis and correlated with SLC7A11 expression in pancreatic cancer.

**Conclusions:**

This study elucidated that LINC00578 acts as an oncogene to promote pancreatic cancer cell progression and suppress ferroptosis by directly combining with UBE2K to inhibit the ubiquitination of SLC7A11, which provides a promising option for the diagnosis and treatment of pancreatic cancer.

## 1. Introduction

Pancreatic cancer is a highly aggressive malignancy worldwide with rapid development and an extremely poor prognosis. It is currently regarded as the fourth leading cause of cancer mortality and is expected to be the second leading cause in 2030 [1]. The high mortality of pancreatic cancer is predominantly due to a lack of screening and diagnosis methods, low resection rates, and insensitivity to chemotherapy and radiotherapy [2]. Therefore, further exploration and understanding of the underlying specific molecular mechanisms of pancreatic cancer progression are of great necessity.

Long noncoding RNAs (lncRNAs) are RNA molecules with transcript lengths longer than 200 nucleotides that are generally considered to be without the potential to encode proteins. Many studies have proven the crucial roles of lncRNAs in modulating biological activities in cancer cells, including cell proliferation, invasion, ferroptosis, and apoptosis [3–5]. Recently, the mechanisms by which lncRNAs fulfill their tumor-suppressing and tumor-driving functions in pancreatic cancer have attracted considerable attention. LINC00578, a newly identified lncRNA, was found to be associated with prognosis in lung cancer and breast cancer [6, 7]. In addition, another study indicated that LINC00578 is upregulated and could be a prognostic signature in pancreatic cancer [8]. Nevertheless, the concrete mechanisms of LINC00578 in pancreatic cancer development and metastasis remain to be elucidated.

Ferroptosis is a novel cell death modality that is triggered by intracellular iron-dependent phospholipid peroxidation. To date, ferroptosis has been found in a variety of cancer cells, including hepatocellular cancer cells, breast cancer cells, and pancreatic cancer cells. Solute carrier family 7 member 11 (SLC7A11), a critical regulator of ferroptosis, functions to import cystine for glutathione biosynthesis and antioxidant defense, thus inhibiting ferroptosis. SLC7A11 overexpression promotes phospholipid peroxidation, which inhibits ferroptosis events in various tumor cells [9–11]. Mounting studies have illustrated that lncRNAs are involved in cancer progression by regulating ferroptosis events. For example, the lncRNA OIP5-AS1-induced inhibitory effect on ferroptosis promotes prostate cancer progression through the miR-128-3p/SLC7A11 pathway [12]. lncRNA MT1DP promotes erastin-induced ferroptosis in lung cancer cells via MT1DP/miR-365a-3p/NRF2 signaling [13]. Intriguingly, chemotherapy-resistant cancer cells, especially those prone to metastasis, are very susceptible to ferroptosis [14]. Notably, although ferroptosis-related lncRNAs have been indicated as prognostic markers in pancreatic cancer, no studies have reported on the role of ferroptosis in the regulation of pancreatic cancer cell progression by lncRNAs.

In our study, the effect of LINC00578 on the development and metastasis of pancreatic cancer was determined. We also explored the role of ferroptosis in regulating pancreatic cancer cell development and invasion by LINC00578. Furthermore, we mechanistically explored whether LINC00578 inhibits ferroptosis in pancreatic cancer cells by reducing ubiquitin-conjugating enzyme 2K- (UBE2K-) mediated SLC7A11 degradation. To conclude, our study will provide vital theoretical evidence for explaining the mechanisms by which LINC00578 inhibits ferroptosis in pancreatic cancer and will provide a novel target for the diagnosis and treatment of pancreatic cancer.

## 2. Materials and Methods

### 2.1. Cell Culture and Treatment

The human pancreatic cancer cell lines PL45 and PATU8988 were delivered from the Chinese Academy of Sciences (Shanghai, China). The PL45 and PATU8988 cell lines were cultured in Dulbecco's modified Eagle's medium (DMEM; HyClone) supplemented with 10% fetal bovine serum (FBS; GIBCO) under a humidified atmosphere of 5% CO_2_ at 37°C. LINC00578 shRNA plasmids, LINC00578 Sh-NC plasmids, LINC00578 overexpression plasmids, LINC00578 vector plasmids, and Si-SLC7A11 plasmids were purchased from GenePharma (Shanghai, China). We treated PL45 cells with lentivirus and selected puromycin. For transient silencing, INTERFERin Reagent was used to transfect siRNA into PATU988 cells. Regarding stable LINC00578 silencing and transient SLC7A11 silencing, the target sequences are listed in Table S1. For the cell death assay, LINC00578-overexpressing cells and vector cells were treated with different stimuli, including erastin (Beyotime, China), ferrostatin-1 (Fer-1) (Beyotime, China), erastin+Fer-1, and DMSO.

### 2.2. Human Tissue Samples

Pancreatic cancer and adjacent nontumor tissue samples in the Su cohort and IHC samples were collected from the Department of General Surgery, the First Affiliated Hospital of Soochow University. Before surgery, none of the patients received preoperative chemotherapy or radiotherapy. The excised samples were rapidly stored in −80°C liquid nitrogen or formalin. The research was approved by the First Affiliated Hospital of Soochow University. All patients signed informed consent forms.

### 2.3. RNA Isolation and Quantitative Real-Time PCR (qRT–PCR)

TRIzol reagent (Invitrogen, USA) was used to extract total RNA. The RNA concentration was then detected. Subsequently, 1 *μ*g of total RNA was reverse transcribed with random primers using the RevertAid™ First Strand cDNA Synthesis Kit and oligo (dT) (GENMED Scientific, Inc., USA). PCR was conducted in a 20 *μ*L PCR containing 1 *μ*L of diluted cDNA. qRT–PCR was performed with a LightCycler 480 II Real-Time PCR system using the SYBR Green method. The PCR primers are listed in Table S2. All qRT–PCR experiments were repeated at least three times.

### 2.4. CCK-8 and Colony Formation Assays

A total of 5000 cells in 100 *μ*L of complete medium were seeded into each well of 96-well culture plates to detect PATU8988 and PL45 cell viability. Next, 10 *μ*L of the Cell Counting Kit-8 (CCK8) was added to each well at 0, 24, and 48 h, and after that, the plates were cultured for 2 h under a humidified atmosphere of 5% CO_2_ at 37°C. The absorbance at 450 nm was measured on a 96-well plate reader (Thermo Fisher Scientific).

For colony formation assays, PATU8988 and PL45 cells (a gradient density of 50 cells/well) were seeded in plates and then incubated for 2–3 weeks until the appearance of colonies. Methanol was used to fix cell colonies, which were then stained with Giemsa. Rate of colony formation = (number of clones/number of cells inoculated) × 100%. The experiment was repeated three times.

### 2.5. Wound-Healing Assay

The invasive ability of the pancreatic cancer cells was tested via wound-healing assay. A 10 *μ*L pipette tube was used to create an acellular area, and the distance of cells migrating to the wounded area was observed after 0 and 48 h via photography. The invasive ability was detected by counting the number of cells that migrated to the original wound. The experiment was repeated three times.

### 2.6. Construction of the Tumor Xenograft Model

In this research, all animal studies were approved by the Institutional Animal Care and Use Committee (IACUC) of Soochow University. Nude mice (female, 6 weeks old, and 18 ± 2 g) were delivered from the Shanghai Experimental Animal Centre (Shanghai, China). A total of 5 × 10^6^ PATU8988 cells transfected with LINC00578 overexpression or control vector were slowly injected into the subcutaneous area of the bilateral flanks of the nude mice (*n* = 6 per group). Body weights and tumor volumes were measured every 3 days. The nude mice were euthanized on day 30 or when they were moribund. Then, the tumors were isolated carefully. Each mouse's tumor was measured through length and width and weighed on the scales. Tumor volume = length × width^2^ × 1/2.

### 2.7. Label-Free Proteomics, GO Terms, and KEGG Pathway Analysis

Cells of the Sh-LINC00578 group and Sh-NC group were collected and then sonicated using an ultrasonic processor (Scientz) in a lysis buffer on ice three times. The samples were centrifuged (12,000 g, 4°C) for 10 min to remove the remaining debris. The supernatant was collected, and then, a BCA kit was used to determine the protein concentration of the Sh-LINC00578 group and Sh-NC group. Dithiothreitol (5 mM, 56°C, and 30 min) was used to reduce the protein solution, and the reduced protein was then alkylated by iodoacetamide (11 mM, in darkness, and 15 min) to obtain the postdigested product.

Triethylammonium bicarbonate (TEAB, 100 mM) was added to the protein samples to keep the urea concentration less than 2 M. Next, the mass ratio of trypsin to protein was maintained at 1 : 50 by adding trypsin to the protein. It was digested overnight. Then, a second 4 h digestion was performed at a 1 : 100 trypsin to protein ratio. Label-free LC–MS/MS analysis was used to analyze collected protein samples from the Sh-LINC00578 group and the Sh-NC group. Tandem mass spectrometry (MS/MS) was performed in Q Exactive™ Plus (Thermo) on peptides previously subjected to an NSI source. The MaxQuant search engine (v.1.5.2.8) was used to further process the MS/MS data. Then, the tandem mass spectra were searched using the human UniProt database, which was concatenated with the reverse decoy database.

Then, GO terms were used to classify the proteins of the Sh-LINC00578 group and the Sh-NC group into three categories: cellular compartment, molecular function, and biological process. For enrichment of the differentially expressed proteins, the evaluation was conducted via Fisher's exact test. A *P* value < 0.05 was considered statistically significant.

The enriched pathways were identified via Kyoto Encyclopedia of Genes and Genomes (KEGG) pathway analysis. For enrichment of the differentially expressed proteins, evaluation was conducted via Fisher's exact test. A *P* value < 0.05 was considered statistically significant.

### 2.8. Western Blot Analysis

The RIPA lysis buffer (Beyotime Biotechnology, Shanghai, China) was used to extract total proteins, which were then quantified by the bicinchoninic acid (BCA) quantitative protein assay according to the manufacturer's instructions. Then, 10% SDS–PAGE was used to separate protein lysates. Proteins were transferred to PVDF membranes. The membrane was blocked with 5% nonfat milk. Primary antibodies were added and incubated at 4°C overnight. Next, the membranes were incubated with horseradish peroxidase-conjugated anti-rabbit IgG or anti-mouse IgG for 1 h, and then, the blots were detected using an enhanced chemiluminescence (ECL) detection system (FDbio, Shanghai, China). GAPDH was chosen as an internal loading control. All primary and secondary antibodies are presented in Table S3. All western blot assays were repeated at least three times.

### 2.9. Measurement of Ferrous Iron (Fe^2+^) and Malondialdehyde (MDA)

Cells were treated according to the corresponding procedures. An Iron Assay Kit (AB83366, Abcam) was used to detect the Fe^2+^ content in PATU8988 and PL45 cells. First, the cells were added to an iron assay buffer, which was then homogenized on ice. Then, the samples were centrifuged at 13,000 × g and 4°C for 10 min. Next, the supernatant was collected. Fifty microliters of supernatant were incubated with 50 *μ*L of assay buffer at room temperature for 30 min. Subsequently, 50 *μ*L of the assay buffer with 200 *μ*L of reagent mix was incubated at room temperature in the dark for 30 min. The absorbance was measured at 593 nm using a microplate reader.

PATU8988 and PL45 cells were plated in 6-well cell culture plates (Corning). After cell homogenization, a BCA protein assay kit (Beyotime) was used to measure protein concentrations, and then, intracellular MDA levels were detected using a lipid peroxidation MDA assay kit (Beyotime). This experiment was repeated at least three times.

### 2.10. Measurement of Reactive Oxygen Species (ROS) and Mitochondrial Membrane Potential (MMP)

To determine ROS levels, vector and OE-LINC00578 group cells were seeded in a 6-well plate. Then, the culture media was replaced with serum-free media containing 10 *μ*mol/L of 2′,7′-dichlorodihydrofluorescein diacetate (Sigma). The samples were then placed in the dark for 20 min and shaken gently every 5 min. The cells were resuspended in PBS and then subjected to flow cytometry to detect changes in ROS expression levels. The experiment was repeated three times.

Tetraethylbenzimidazolylcarbocyanine iodide (JC-1) staining was used to measure MMP in PATU8988 and PL45 cells. Cells were treated with NaAsO_2_ and stained with 5 *μ*M JC-1 (Beyotime, China) at 37°C in the dark for 20 min. The cells were washed twice with PBS and observed using a fluorescence microscope. The ratio of red/green fluorescence intensity was analyzed via Image-Pro Plus 6.0 software.

### 2.11. RNA Pull-Down and Mass Spectrometry

A DNA fragment containing LINC00578 full-length sequence or a negative control sequence was PCR amplified using SP6 (for antisense)/T7 (for sense) polymerase. Biotin RNA Labeling Mix (Roche) and T7 RNA polymerase were used for the reverse transcription of biotin-labeled RNA. Then, the products were treated with RNase-free DNase I (Roche) and an RNeasy Mini Kit (Qiagen, MD, USA) for purification. Four micrograms of RNA were denatured in a PA buffer. The mixture was then cooled to room temperature. Subsequently, the folded RNA was incubated with streptavidin Dynabeads (Invitrogen) for 1 h at 4°C with 2 U/mL RNasin (Promega). The cells were washed to clear the protein lysate from 1 × 10^7^ PL45 cells, which were then incubated with the folded RNA-bead complex with 20 *μ*g/mL yeast tRNA. The beads were boiled with 40 *μ*L of 1x SDS loading buffer after washing. Sodium dodecyl sulfate–polyacrylamide gel electrophoresis can further separate lncRNA-interacting proteins. The gel was stained with silver. Next, the specific bands of LINC00578 were subjected to Q Exactive mass spectrometry.

For each sample, 1/2 peptides were alienated and explored using Q Exactive mass spectrometry (Thermo). Then, the peptides were separated. H_2_O with 0.1% FA, 2% ACN (phase A) and 80% ACN, and 0.1% FA (phase B) were the mobile phases. A 120 min gradient at a 300 nL/min flow rate was performed to separate the samples. The gradient was comprised of an increase of solvent B from 0% to 3% in 8 min, 3% to 8% in 3 min, 8% to 20% in 77 min, 20% to 40% in 10 min, and 40% to 90% in 4 min, then holding at 90% for 6 min, and a subsequent decrease of solvent B from 90% to 3% in 4 min and 3% to 0% in 8 min.

Data-dependent acquisition was accomplished in profile as well as in positive mode with an Orbitrap analyzer at a resolution of 70,000 FWHM and a *m*/*z* range of 300-1400 for MS1. For dd-MS2, the resolution was fixed to 17,500 FWHM. The automatic gain control (AGC) target for MS1 was 3.0 e^6^ with a max IT of 60 ms and was 5.0 e^4^ for dd-MS2 with a max IT of 80 ms. HCD with a normalized collision energy (NCE) of 27% was used to disintegrate the top 10 strongest ions.

Then, we investigated the raw MS files with Proteome Discoverer. The protein sequence database (UniProt_Human_2020_08.13) was acquired from UniProt. All the additional parameters were kept as default.

### 2.12. RNA Immunoprecipitation (RIP) Assay

A RIP RNA-Binding Protein Immunoprecipitation Kit (Millipore, MA) was used to perform RIP. In brief, 2 × 10^7^ PL45 cell lysates were incubated with beads conjugated with anti-UBE2K or negative control (normal mouse IgG). The immunoprecipitated RNAs were extracted. Then, qRT–PCR was performed to verify binding target enrichment. Finally, the products were subjected to agarose gel electrophoresis. The primers for LINC00578 are listed in Table S2. The RIP assay was repeated three times.

### 2.13. Immunoprecipitation Assay

The cells were washed with PBS and lysed with a protein extraction reagent buffer on ice for 1 h. The samples were centrifuged at 15,000 × g at 4°C for 10 min, and then, the supernatant was collected. The supernatant was incubated with a primary antibody against GAPDH (1 : 2000; 60004-1-Ig, Proteintech) or mouse immunoglobulin G control at 4°C for 4 h with gentle agitation. Regarding the coimmunoprecipitations (IPs), 20 *μ*L of prewashed protein A/G agarose beads was incubated with 400 *μ*g of samples at 4°C overnight with gentle agitation. After incubation, the immune complexes were washed three times with a lysis buffer. Then, the proteins were collected after centrifugation and boiled with the SDS loading buffer. Finally, western blotting was performed. The experiment was repeated three times.

### 2.14. Immunohistochemical (IHC) Assay

Tissues were fixed with formalin and embedded in paraffin. Four-micrometer-thick sections were cut and mounted on glass slides according to the specifications of the S-P (streptavidin peroxidase) kit. They were incubated with anti-SLC7A11 (1 : 200; 26864-1-AP, Proteintech) antibodies overnight at 4°C. Subsequently, the membranes were incubated with the secondary antibody and ExtrAvidin-conjugated horseradish peroxidase. Sections were evaluated via light microscopy.

### 2.15. Bioinformatics

Bioinformatic analysis was carried out with pancancer and pancreatic cancer data, which were from The Cancer Genome Atlas (TCGA). The data were analyzed by the R package (Version 4.2.0). Kaplan–Meier analysis was conducted to construct overall survival curves.

### 2.16. Statistical Analysis

SPSS 21.0 software (IBM, Armonk, NY, USA) was used for statistical analysis. All continuous data are presented as the mean ± standard deviation for multiple (SD). GraphPad Prism 9 software was used for statistical analysis. For cellular assays in vitro and animal experiments in vivo, a two-sided Student's *t* test was performed to analyze the difference between two groups. One-way ANOVA was performed for comparisons among multiple groups. The correlation between LINC00578 expression and clinical characteristics was validated by Fisher's exact test. In this study, a *P* value < 0.05 was defined as significantly different.

## 3. Results

### 3.1. The Expression of LINC00578 Is Closely Correlated with Clinicopathologic Factors and Associated with Poor Prognosis in Pancreatic Cancer

**Figure 1:**
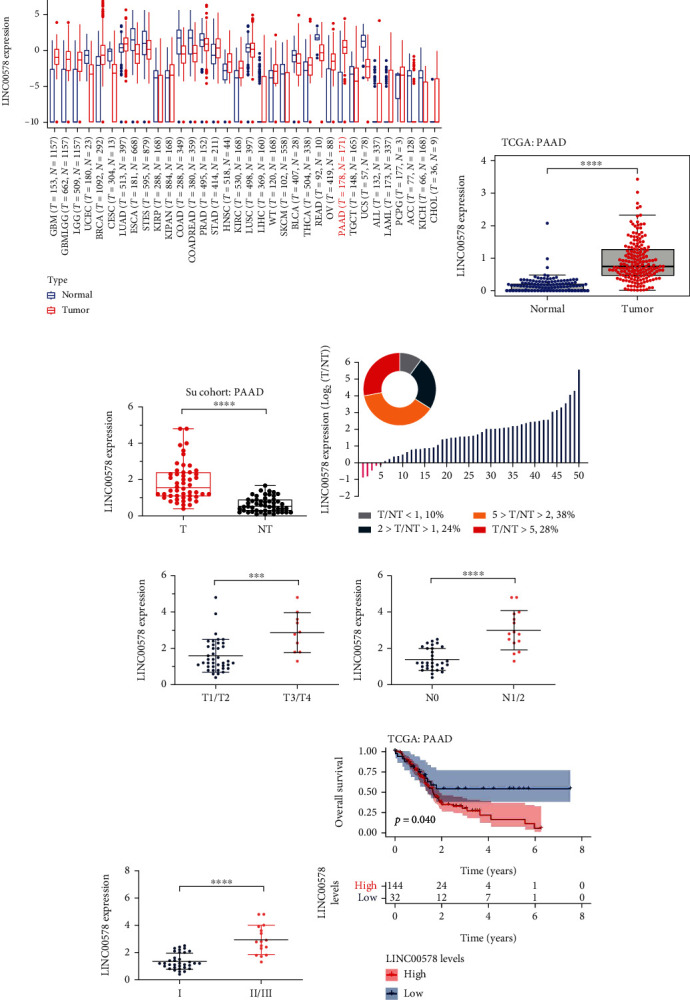
LINC00578 is upregulated in pancreatic cancer and correlated with clinicopathological factors. (a) Box plot of LINC00578 expression in 34 kinds of cancers from the TCGA (PANCAN, *N* = 19131, *T* = 60499) cohort. (b) Box plot of LINC00578 expression in both pancreatic cancer tissues (*n* = 177) and normal tissues (*n* = 171) from the TCGA-GTEx cohort. (c) Box plot of LINC00578 expression in pancreatic cancer tissues (*n* = 50) and adjacent normal tissues (*n* = 50) from the Su cohort. (d) The expression ratio of LINC00578 between tumors and adjacent normal tissues from the Su cohort. (e) The expression of LINC00578 between T1/T2 and T3/T4 stages from the Su cohort. (f) The expression of LINC00578 between N0 and N1/N2 stages from the Su cohort. (g) The expression of LINC00578 between Grade I and Grade II from the Su cohort. (h) Kaplan–Meier curve showing the correlation of the LINC00578 expression with overall survival (OS) (*P* = 0.04) in the TCGA cohort. ^∗^*P* < 0.05; ^∗∗^*P* < 0.01; ^∗∗∗^*P* < 0.001; ^∗∗∗∗^*P* < 0.0001. Abbreviations: ACC: adrenocortical carcinoma; ALL: acute lymphoblastic leukemia; BLCA: bladder urothelial carcinoma; CESC: cervical squamous cell carcinoma and endocervical adenocarcinoma; COAD: colon adenocarcinoma; COADREAD: colon adenocarcinoma/rectum adenocarcinoma; CHOL: cholangiocarcinoma; ESCA: esophageal carcinoma; GBM: glioblastoma multiforme; GBMLGG: glioma; HNSC: head and neck squamous cell carcinoma; KICH: kidney chromophobe; KIRC: kidney renal clear cell carcinoma; KIRP: kidney renal papillary cell carcinoma; KIPAN: pankidney cohort (KICH+KIRC+KIRP); LAML: acute myeloid leukemia; LGG: brain lower grade glioma; LIHC: liver hepatocellular carcinoma; LUAD: lung adenocarcinoma; LUSC: lung squamous cell carcinoma; OV: ovarian serous cystadenocarcinoma; PAAD: pancreatic adenocarcinoma; PCPG: pheochromocytoma and paraganglioma; PRAD: prostate adenocarcinoma; READ: rectum adenocarcinoma; STAD: stomach adenocarcinoma; STES: stomach and esophageal carcinoma; SKCM: skin cutaneous melanoma; THCA: thyroid carcinoma; TGCT: testicular germ cell tumors; UCEC: uterine corpus endometrial carcinoma; UCS: uterine carcinosarcoma; WT: high-risk Wilms tumor.

To grasp the role of LINC00578 in cancer progression, pancancer analysis was first performed in a TCGA cohort, and the results showed that the expression of LINC00578 was dramatically decreased in 15 kinds of cancers, including colon adenocarcinoma and esophageal carcinoma, and increased in 16 kinds of cancers, including pancreatic adenocarcinoma and liver hepatocellular carcinoma (Figure 1(a)). Subsequently, we analyzed LINC00578 expression levels in a PAAD cohort from the TCGA database. As expected, the expression level of LINC00578 was increased in pancreatic cancer compared with normal tissues (Figure 1(b)). Therefore, we focused on investigating the role of LINC00578 in pancreatic cancer. Subsequently, the expression of LINC00578 in the Su cohort of 50 paired cases was examined to validate the increased level of LINC00578 in pancreatic cancer, and the results showed that the expression level of LINC00578 was significantly higher in pancreatic cancer tissues than in benign tissues (Figure 1(c)). The expression ratio of LINC00578 between tumors and adjacent normal tissues showed that the ratio of T/NT of 38% of cases ranged from 2 to 5, and the ratio of 28% of cases exceeded 5 (Figure 1(d)). Then, to determine whether LINC00578 expression is associated with pancreatic cancer progression, the relationship between LINC00578 expression and clinicopathologic factors was analyzed, and we discovered that a higher LINC00578 expression level was positively associated with advanced T stage (*P* = 0.0106), N stage (*P* = 0.0001), and TNM stage (*P* < 0.0001) (Figures 1(e)–1(g), Table 1). Kaplan–Meier analysis of PAAD cohorts in TCGA showed that a high LINC00578 expression level predicted poor overall survival of patients with pancreatic cancer (Figure 1(h), Figure S1).

### 3.2. LINC00578 Promotes Pancreatic Cancer Cell Proliferation and Invasion In Vitro and Tumor Growth In Vivo

**Figure 2:**
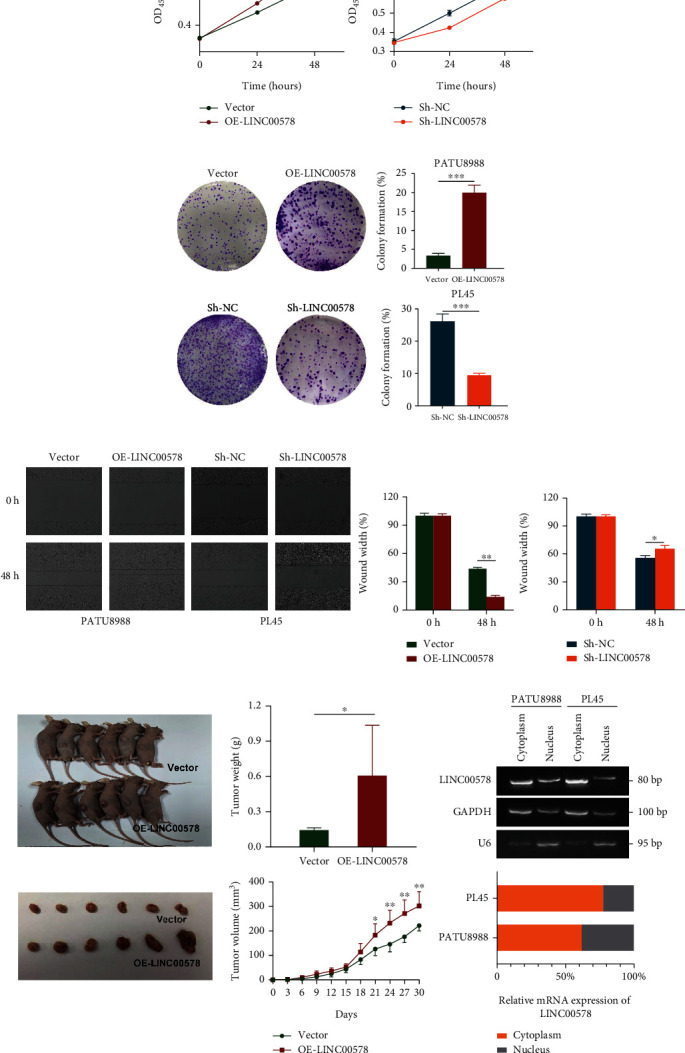
LINC00578 promotes pancreatic cancer cell proliferation and invasion in vitro and tumor growth in vivo. (a) CCK-8 assays were applied to detect cell viability at the indicated time points in PATU8988 cells transfected with LINC00578 (OE-LINC00578) versus empty vector-transfected PATU8988 cells (Vector) and LINC00578 knockdown PL45 cells (Sh-LINC00578) versus empty vector knockdown PL45 cells (Sh-NC). (b) Colony formation assays were applied to detect cell proliferation in PATU8988 cells (OE-LINC00578 group versus vector group) and PL45 cells (Sh-LINC00578 group versus Sh-NC group). (c) Wound-healing assay indicating LINC00578-induced increased migration in PATU8988 and PL45 cells. (d) Tumor size, volume, and weight at day 30 after subcutaneous injection of LINC00578-overexpressing PATU8988 cells (*n* = 6) compared with the vector group (*n* = 6). (e) A nucleocytoplasmic separation assay was used to determine the intracellular location of LINC00578 in PATU8988 cells and PL45 cells. ^∗^*P* < 0.05; ^∗∗^*P* < 0.01; ^∗∗∗^*P* < 0.001.

To identify the biological role of LINC00578 in pancreatic cancer, we first upregulated the expression of LINC00578 in the PATU8988 cell line and downregulated the expression of LINC00578 in the PL45 cell line using lentivirus stable transfection. The overexpression and knockdown efficiency of OE-LINC0578 and Sh-LINC00578 are shown in Figure S2. Next, the possible role of LINC00578 in regulating pancreatic cancer cell growth and invasion was evaluated by CCK-8, colony formation, and wound-healing assays. LINC00578-overexpressing cells (OE-LINC00578) showed significantly increased proliferation, colony formation, and invasion ability compared with empty vector-transfected cells (Vector) and, as expected, compared with the Sh-NC group; notably, decreased proliferation, colony formation, and invasion ability were observed in LINC00578 knockdown cells (Sh-LINC00578) (Figures 2(a)–2(c)). Then, to investigate whether LINC00578 can regulate pancreatic cancer growth in vivo, LINC00578-overexpressing cells or empty vector-transfected cells were injected subcutaneously into nude mice. The results showed that the tumor weight and volume were greater in the OE-LINC00578 group than in the vector group (Figure 2(d)). Furthermore, a nucleocytoplasmic separation experiment revealed the cytoplasmic distribution of LINC00578, indicating posttranslational regulation pathways (Figure 2(e)). Collectively, our data demonstrate that LINC00578 can promote the proliferative and invasive abilities of pancreatic cancer cells.

### 3.3. LINC00578 Represses Ferroptosis in Pancreatic Cancer

**Figure 3:**
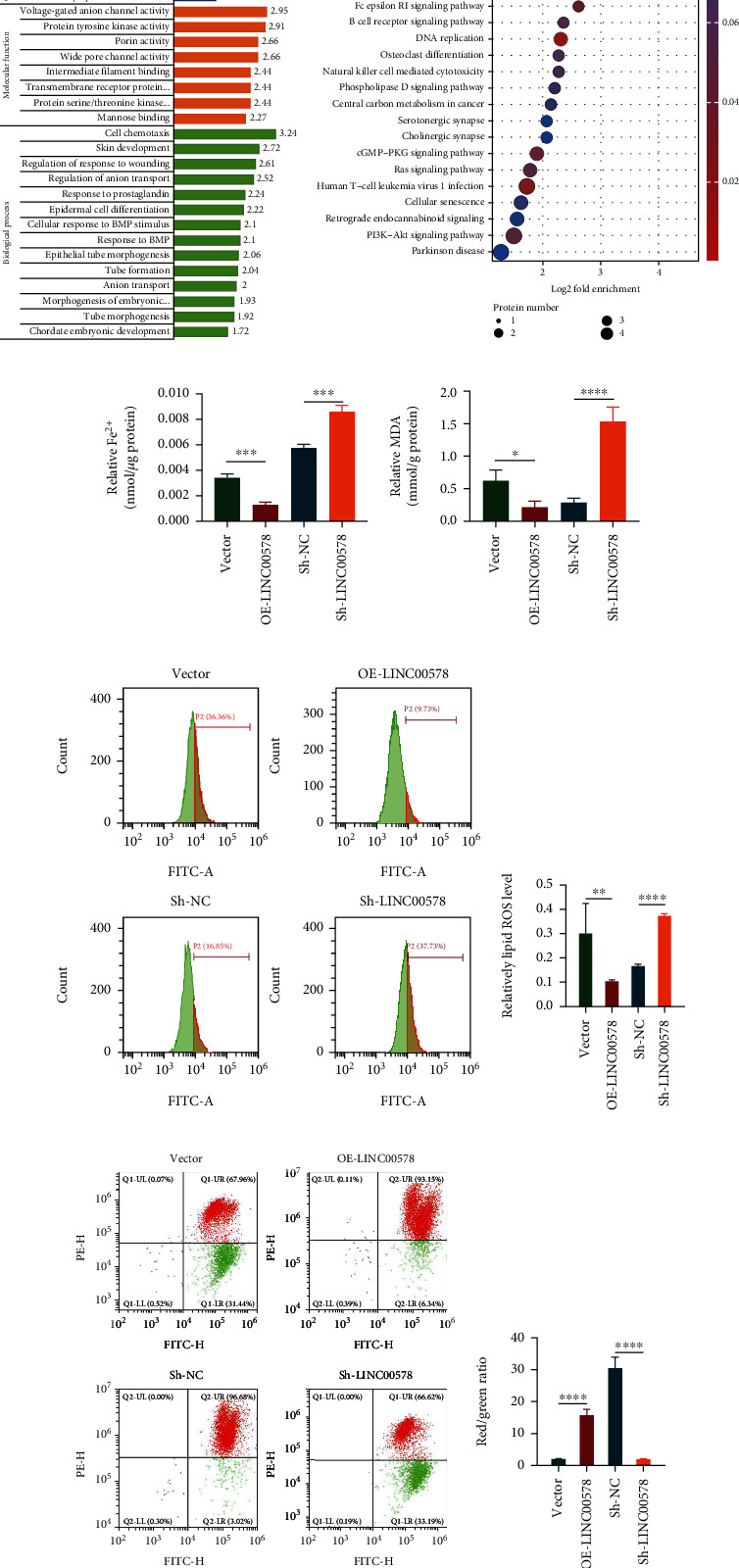
LINC00578 acts as a ferroptosis repressor in pancreatic cancer. (a) Statistical distribution of differentially expressed proteins in GO secondary classification. Blue represents cellular components. Yellow represents molecular function. Green represents biological processes. (b) Bubble map showing the distribution of differentially expressed proteins (Sh-LINC00578 group versus Sh-NC group, selected by label-free proteomics) enriched in the KEGG pathway. (c) Quantitative analysis of intracellular Fe^2+^. (d) Quantitative analysis of intracellular malondialdehyde (MDA). (e) OE-LINC00578, vector, Sh-LINC00578, and Sh-NC cells were stained with DCFH-DA to measure intracellular ROS levels via flow cytometry. (f) Quantitative analysis of ROS generation. (g) OE-LINC00578, vector, Sh-LINC00578, and Sh-NC cells were stained with JC-1 to measure MMP levels via flow cytometry. Green represents the depolarized state of cells. Red represents the normal state of cells. (h) Quantitative analysis of MMP. Red/green ratio showing the effect of LINC00578 on inhibiting MMP depolarization. ^∗^*P* < 0.05; ^∗∗^*P* < 0.01; ^∗∗∗^*P* < 0.001; ^∗∗∗∗^*P* < 0.0001.

To further investigate the effect of LINC00578 expression on related proteins, label-free proteomic analysis was performed. Compared with the Sh-NC group, there were 43 upregulated proteins and 32 downregulated proteins in the Sh-LINC00578 group (Figure S3). Gene Ontology analysis revealed that LINC00578 was correlated with voltage-gated anion channel activity and transmembrane receptor proteins in molecular function and associated with cell chemotaxis and regulation of anion transport in biological processes (Figure 3(a)). The results of KEGG analysis of these proteins indicated that proteins with a trend of change when LINC000578 was knocked down were significantly correlated with ferroptosis (Figure 3(b)). We hypothesized that LINC00578 is associated with ferroptosis in pancreatic cancer. Ferroptosis is characterized by the accumulation of iron-dependent lipid peroxidative products and increasing levels of ROS. To verify our hypothesis, we first detected the Fe^2+^ content in PATU8988 cells and PL45 cells, and the results showed that the Fe^2+^ content was significantly decreased in the OE-LINC00578 group compared to the vector group and increased in the Sh-LINC00578 group compared to the Sh-NC group (Figure 3(c)). Meanwhile, MDA, the end product of lipid peroxidation, was markedly decreased in the LINC00578-overexpressing group and increased in the LINC00578 knockdown group (Figure 3(d)). Next, we detected the ROS level through flow cytometry. As expected, LINC00578-overexpressing cells showed decreased ROS levels compared with the vector group, while LINC00578 knockdown cells showed increased ROS levels compared with the Sh-NC group (Figures 3(e) and 3(f)). Given that cysteine deprivation-induced ferroptosis leads to MMP depolarization, MMP assays were then performed. As shown in Figures 3(g) and 3(h), MMP was increased in the LINC00578-overexpressing group and decreased in the LINC00578 knockdown group.

**Figure 4:**
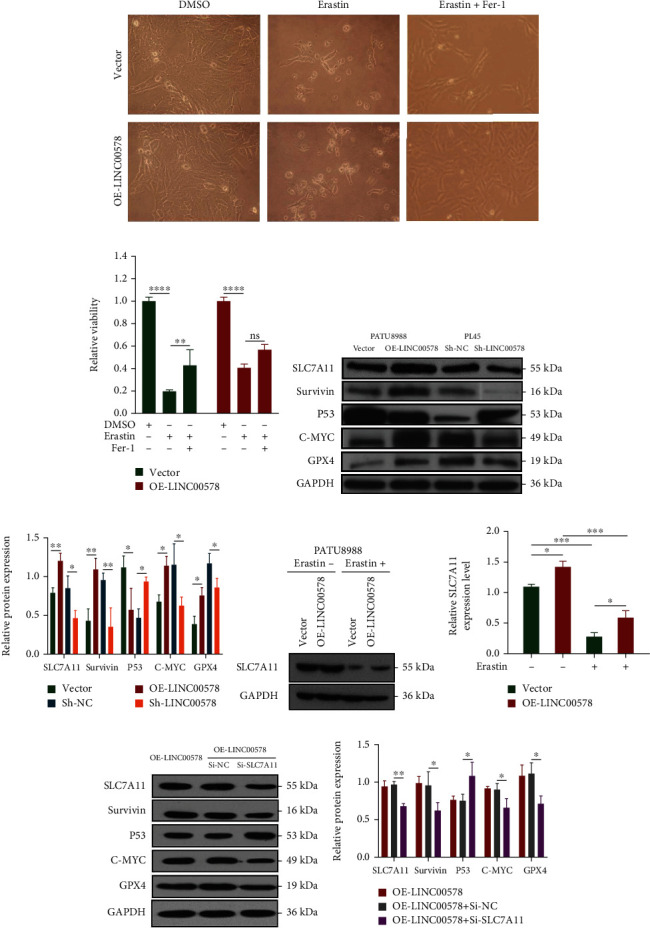
LINC00578 negatively modulates ferroptosis by targeting SLC7A11. (a) Observation of cell number changes in the OE-LINC00578 group versus the vector group under the induction of DMSO, erastin, and erastin+Fer-1. (b) CCK-8 assay showing the relative cell viability in the OE-LINC00578 group versus the vector group after induction with DMSO, erastin, and erastin+Fer-1. (c) Western blot results showing the levels of ferroptosis-related proteins (SLC7A11, P53, GPX4, Survivin, and GPX4) in pancreatic cancer cells. (d) Quantitative analysis of the levels of ferroptosis-related proteins. (e) Western blotting to measure the protein level of SLC7A11 in PATU8988 cells with or without erastin induction. (f) Quantitative analysis of the protein level of SLC7A11. (g) SLC7A11 silencing inhibited LINC00578-induced ferroptosis-related protein (SLC7A11, P53, GPX4, Survivin, and GPX4) expression. (h) Quantitative analysis of the protein level of LINC00578-induced ferroptosis-related proteins (SLC7A11, P53, GPX4, Survivin, and GPX4) reversed by SLC7A11 silencing. ^∗^*P* < 0.05; ^∗∗^*P* < 0.01; ^∗∗∗^*P* < 0.001 and ^∗∗∗∗^*P* < 0.0001.

To verify the repressor actor of LINC0578 in ferroptosis, we observed the cell number changes of LINC00578-overexpressing and vector groups induced by erastin and Fer-1. The results showed that LINC00578 overexpression significantly weakened erastin-induced decreased viability. In addition, the ferroptosis inhibitor Fer-1 rescued the erastin-induced decrease in viability in the vector group, which was not obvious in the OE-LINC00578 group (Figures 4(a) and 4(b)). These findings indicated that the ferroptosis activator erastin could inhibit the viability of PATU8988 cells and that LINC00578 overexpression inhibited erastin-induced decreased viability in PATU8988 cells.

### 3.4. LINC00578 Negatively Modulates Ferroptosis by Targeting SLC7A11

**Figure 5:**
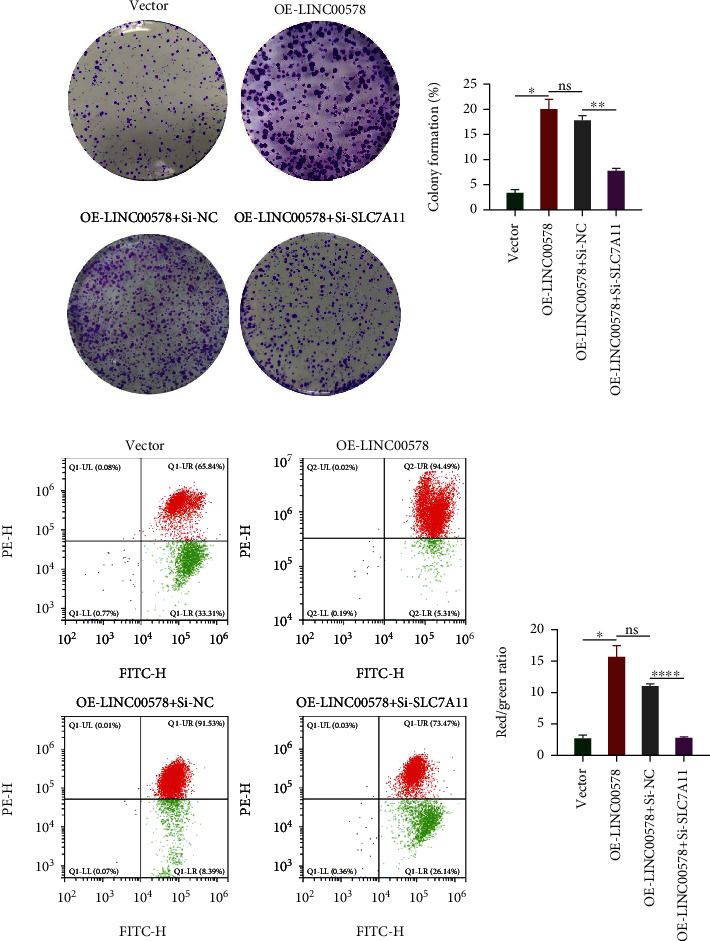
SLC7A11 knockdown reversed the effects of OE-LINC00578 on ferroptosis. (a) Colony formation assay showed that cell proliferation promotion in PATU8988 cells by OE-LINC00578 introduction was reversed by Si-SLC7A11. (b) Quantitative analysis showed that the promotion of cell proliferation in PATU8988 cells by OE-LINC00578 introduction was reversed by Si-SLC7A11 by the colony formation assay. (c) Si-LINC00578 significantly reversed the inhibition of MMP depolarization in PATU8988 cells by OE-LINC00578 introduction. (d) Quantitative analysis of MMP. Red/green ratio showing the effect of Si-SLC7A11 in the reverse of OE-LINC00578 on inhibiting MMP depolarization. ^∗^*P* < 0.05; ^∗∗^*P* < 0.01; ^∗∗∗∗^*P* < 0.0001.

To understand the mechanism by which LINC00578 modulates ferroptosis, the expression levels of ferroptosis-related proteins in response to overexpression or knockdown of LINC00578 were detected. Compared to the vector group, the protein levels of SLC7A11, Survivin, C-MYC, and GPX4 increased, while the protein levels of P53 decreased in the LINC00578-overexpressing group. Meanwhile, compared with the Sh-control group, the protein levels of SLC7A11, Survivin, C-MYC, and GPX4 decreased, while the protein levels of P53 increased in response to Sh-LINC00578 (Figures 4(c) and 4(d)). Subsequently, we detected whether the protein levels of SLC7A11 changed after the addition of erastin. As shown in Figures 4(e) and 4(f), LINC00578 promoted the protein levels of SLC7A11, and as expected, erastin repressed the protein levels of SLC7A11 in both the LINC00578-overexpressing and vector groups. To further validate whether LINC00578 modulates ferroptosis via SLC7A11, several rescue experiments were performed. SLC7A11 was knocked down through siRNA. Compared to the OE-LINC00578+Si-NC group, the protein levels of SLC7A11, Survivin, C-MYC, and GPX4 decreased, while P53 increased in the OE-LINC00578+Si-SLC7A11 group in response to the knockdown of SLC7A11 (Figures 4(g) and 4(h)). Colony formation assays showed that LINC00578 overexpression enhanced the proliferative ability of PATU8988 cells and that Si-SLC7A11 reversed this effect (Figures 5(a) and 5(b)). In the MMP experiment, Si-SLC7A11 successfully rescued the inhibition of mitochondrial membrane potential depolarization mediated by OE-LINC00578 in the PATU8988 cell line (Figures 5(c) and 5(d)). These findings illustrate that LINC00578 suppresses ferroptosis in pancreatic cancer cells primarily through SLC7A11.

### 3.5. LINC00578 Inhibits SLC7A11 Ubiquitination by Interacting with UBE2K

**Figure 6:**
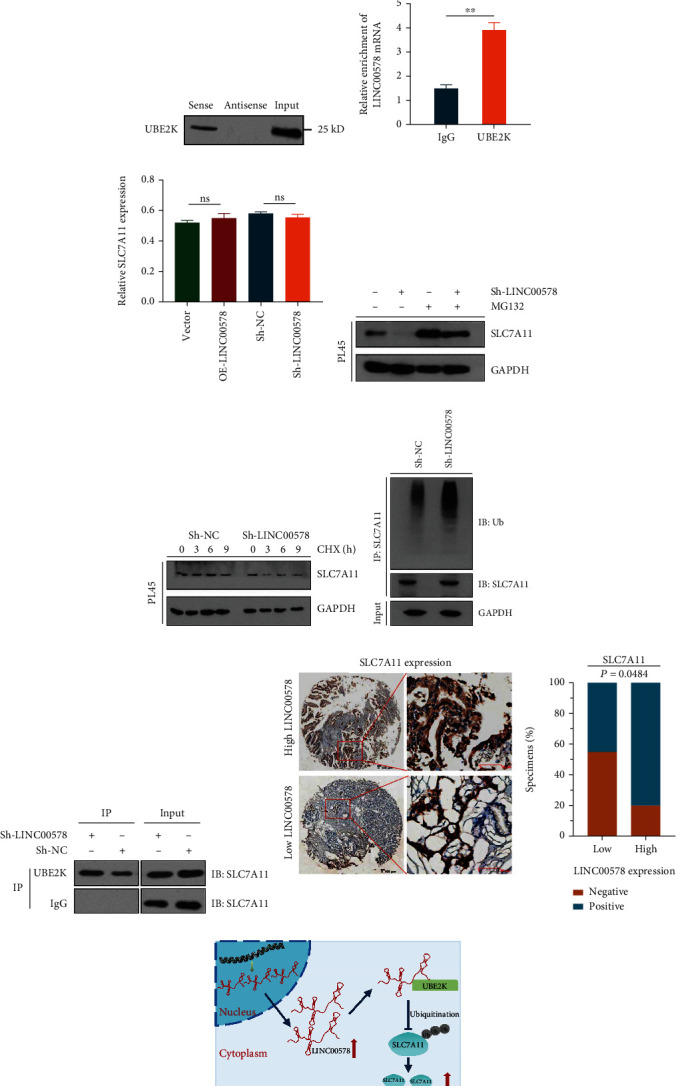
LINC00578 inhibits the ubiquitination of SLC7A11 by directly interacting with UBE2K. (a) Venn diagram showing 5 LINC00578 antisense proteins and 18 LINC00578 sense proteins by pull-down assay of PL45 cells. (b) Western blotting showing that LINC00578 interacts with UBE2K in pancreatic cancer cells. (c) qRT–PCR showing LINC00578 enrichment in UBE2K-immunoprecipitated RNAs in PL45 cells. An antibody against human IgG was used as a negative control for RIP. (d) qRT–PCR analysis showing the RNA level of SLC7A11 in the OE-LINC00578 group versus the vector group and the Sh-LINC00578 group versus the Sh-NC group. (e) Western blot assay revealing the reverse effect of MG132 on the protein degradation of SLC7A11 by Sh-LINC00578 introduction. (f) Western blot assay showing the promotion of CHX on the protein degradation rate of SLC7A11 by Sh-LINC00578 introduction. (g) Cells stably expressing Sh-NC or Sh-LINC00578 were subjected to a deubiquitination assay, and polyubiquitylated proteins were detected with an anti-Ub antibody. (h) Co-IP assay showing the interaction between UBE2K and SLC7A11. (i) IHC for SLC7A11 was applied between the LINC00578 high-expression group (above panel) and the low-expression group (below panel) in 50 paired pancreatic cancer tissue samples. The correlation analysis showed a positive correlation between LINC00578 and SLC7A11 (*P* = 0.0484, Fisher's exact test) expression. (j) Diagram illustrating how LINC00578 promotes SLC7A11 to inhibit ferroptosis. ^∗∗^*P* < 0.01.

Then, we wondered how LINC00578 promotes SLC7A11 expression to inhibit ferroptosis in pancreatic cancer. First, an RNA pull-down assay was conducted. There were 5 differentially expressed proteins in the LINC00578-antisense group and 18 differentially expressed proteins in the LINC00578-sense group, among which UBE2K is a ubiquitin-conjugating enzyme and was selected as a candidate target of LINC00578 (Figure 6(a)). Subsequently, we performed a western blot assay to confirm that UBE2K was a specific binding protein for LINC00578 (Figure 6(b)). RIP assays were performed to further verify the specific interaction between LINC00578 and SLC7A11 (Figure 6(c)). The RNA levels of SLC7A11 in the OE-LINC00578/Sh-LINC00578 and vector groups were detected, and there was no significant difference in the RNA levels between the OE-LINC00578/Sh-LINC00578 group and the vector group, indicating that LINC00578 may regulate SLC7A11 through posttranslational pathways (Figure 6(d)).

To validate whether LINC00578 can affect the protein degradation rate of SLC7A11, the stability of SLC7A11 in response to knockdown of LINC00578 was detected with MG132 (proteasome inhibitor) and cycloheximide (protein synthesis inhibitor). As shown in Figure 6(e), treatment with MG132 in the LINC00578 knockdown group increased the expression of SLC7A11. Moreover, LINC00578 knockdown accelerated the protein degradation of SLC7A11 in PL45 cell lines (Figure 6(f)). Then, the SLC7A11 protein was immunoprecipitated in the Sh-LINC00578 and Sh-control groups to investigate the status of SLC7A11 ubiquitination. The results showed a significant increase in ubiquitinated proteins in response to knockdown of LINC00578 (Figure 6(g) and Figure S4). Therefore, we deduced that LINC00578 impeded the ubiquitination of SLC7A11, which inhibits ferroptosis in pancreatic cancer. Next, coimmunoprecipitation (Co-IP) was used to validate the interaction between UBE2K and SLC7A11. The Co-IP results indicated that compared with the Sh-NC group, SLC7A11 was more enriched in the immunoprecipitated products of UBE2K in the Sh-LINC00578 group, proving that LINC00578 knockdown facilitated the interaction between UBE2K and SLC7A11 (Figure 6(h)). Collectively, LINC00578 directly binds UBE2K and affects the interaction between UBE2K and SLC7A11, thereby inhibiting ubiquitination of SLC7A11.

To investigate whether LINC00578 and SLC7A11 are clinically relevant, IHC for SLC7A11 was performed on 50 pancreatic cancer tissues.  Figure 6(i) illustrates that SLC7A11 expression positively correlates with that of LINC00578 (*P* = 0.0484). Collectively, these findings illustrate that LINC00578 binds UBE2K to inhibit ubiquitination of SLC7A11, thus inhibiting ferroptosis in pancreatic cancer, which renders tumor cells highly invasive and aggressive (Figure 6(j)).

## 4. Discussion

Pancreatic cancer is one of the leading causes of cancer-related death worldwide. Moreover, the mortality and incidence of pancreatic cancer are increasing rapidly [15]. Although enormous efforts have been made to investigate early diagnosis and treatment methods, early diagnosis and efficient therapy of pancreatic cancer remain a formidable challenge. More than 90% of the genome is transcribed into ncRNAs, among which lncRNAs constitute the primary elements [16]. lncRNAs have been found to be correlated with various biological activities, including tumorigenesis, inflammation, and cell differentiation, through transcriptional regulation or posttranslational regulation [17–19]. In the nucleus, lncRNAs participate in alternative splicing and transcriptional regulation and control the epigenetic state of genes. In the cytoplasm, the regulatory effect of lncRNAs on gene expression is mostly at the posttranslational level, including stabilizing mRNAs and competing for microRNA-mediated inhibition [20, 21]. In this study, we investigated a novel lncRNA named LINC00578 in pancreatic cancer, which was upregulated in pancreatic cancer cell lines and tissues and correlated with poor patient survival. In addition, LINC00578 was found to be related to the advanced clinical stage in pancreatic cancer patients. These findings indicate that LINC00578 might be involved in pancreatic cancer progression. As expected, we demonstrated that LINC00578 is positively regulated in cell proliferation and invasion in vitro and tumorigenesis in vivo in pancreatic cancer. Therefore, LINC00578 plays a positive regulatory role in pancreatic cancer progression.

Ferroptosis, a novel form of iron-dependent cell death due to phospholipid peroxidation, has attracted enormous attention worldwide [22, 23]. Extensive studies have identified that ferroptosis plays a crucial role in regulating tumor progression and maintains promising prospects for cancer therapy [24, 25]. Ferroptosis can be regulated by multiple essential factors, including SLC7A11, GPX4, and P53 [26, 27]. SLC7A11, as a key repressor of ferroptosis, is upregulated in tumors and can interact with ALOX12 to promote tumor progression [28]. He et al. found that as the core target of ferroptosis regulation, SLC7A11 is upregulated in most pancreatic cancer cell lines [29]. However, until now, no study has reported specific regulatory mechanisms between lncRNAs and ferroptosis in pancreatic cancer. Interestingly, our study found that activated LINC00578 can obviously inhibit ferroptosis events, including erastin-induced cell proliferation inhibition, ROS generation, and MMP depolarization. The inhibitory effect of LINC00578 on ferroptosis events can be rescued by SLC7A11 knockdown. Importantly, we first demonstrated that LINC00578 overexpression can inhibit ferroptosis by targeting SLC7A11 in pancreatic cancer, suggesting that LINC00578 regulates pancreatic cancer progression by inhibiting SLC7A11-dependent ferroptosis.

The ubiquitin–proteasome pathway is a prevalent form of endogenous protein degradation, where ubiquitination is mediated by linking ubiquitin to target proteins through an enzymatic reaction. Mounting studies illustrate that cytoplasmic-located lncRNAs are suitable for translational regulation, including ubiquitin-mediated protein stability control and translational efficiency regulation [30–32]. For example, LINC00673 enhances the interaction of PTPN11 with the ubiquitin ligase PRPF19 and promotes PTPN11 degradation through ubiquitination in pancreatic cancer [33]. LINC00669, which is located in the cytoplasm of nasopharyngeal carcinoma cells, can bind to SOC1 and block its ubiquitination of STATA1 [34]. In agreement with these findings, in our study, the direct interaction between cytoplasmic LINC00578 and the ubiquitin regulatory enzyme UBE2K was investigated by a pull-down assay and validated by subsequent western blotting and RIP, revealing that LINC0578 can directly bind UBE2K to control downstream protein stability. SLC7A11 can be regulated at multiple levels, in which posttranslational regulation plays a critical role. For example, Liu et al. identified that OTUB1 (an ovarian tumor family member deubiquitylase) can interact with SLC7A11 to prevent its degradation [35]. Additionally, Chen's study revealed that fascin directly interacts with SLC7A11 and decreases its stability via ubiquitin-mediated degradation [36]. In concert with these findings, we demonstrated that LINC00578 knockdown can promote ubiquitination of SLC7A11. In addition, for the first time to our knowledge, the interaction between UBE2K and SLC7A11 was validated via Co-IP in our study. Considering the direct interaction between LINC00578 and UBE2K that was proven previously, we demonstrated that LINC00578 can directly bind UBE2K, thus reducing UBE2K-mediated ubiquitination of SLC7A11.

Taken together, this study revealed that LINC00578 promoted pancreatic cancer progression and inhibited ferroptosis by directly binding UBE2K to suppress SLC7A11 ubiquitination. However, it is elusive whether LINC00578 participates in pancreatic cancer progression through SLC7A11-independent ferroptosis. Another limitation of this research lies in the cohort sample size, which will be improved in further studies.

## 5. Conclusions

This study illustrates that LINC00578 promotes pancreatic cancer cell progression and suppresses ferroptosis by directly binding UBE2K to inhibit the ubiquitination of SLC7A11. These findings demonstrate that LINC00578 might be a crucial biomarker for pancreatic cancer progression and may provide a promising option for the diagnosis and treatment of pancreatic cancer.

## Figures and Tables

**Table 1:** Relationship between LINC00578 expression level and clinicopathologic factors in pancreatic cancer patients.

Clinicopathologic factors	Cases	LINC00578		*P* value
		Low expression	High expression	
Gender	Male	11	15	0.3961
	Female	14	10	
Age	≥65	13	10	0.5709
	<65	12	15	
T stage	T1+T2	24	16	0.0106^∗^
	T3+T4	1	9	
N stage	N0	24	11	0.0001^∗∗∗^
	N1/2	1	14	
TNM stage	I	24	10	<0.0001^∗∗∗∗^
	II/III	1	15	
Tumor location	Head and neck	11	14	0.5721
	Body and tail	14	11	

^∗^ *P* < 0.05; ^∗∗∗^*P* < 0.001; ^∗∗∗∗^*P* < 0.0001.

## Data Availability

The data are presented within the paper. The raw data supporting the conclusions of this article will be made available on request from the corresponding authors, without undue reservation.

## Supplementary Materials

Supplementary 1 Figure S1: Kaplan-Meier analysis of the correlation of LINC00578 expression with disease-free survival (DFS) (*P* = 0.002), from the cBioPortal (TCGA Firehose Legacy) cohort. Figure S2: relative expression levels of LINC00578 in PATU8988 cells with LINC00578 overexpression and PL45 cells with LINC00578 knockdown. Figure S3: differential expressed proteins between Sh-LINC00578 and Sh-NC groups selected by label-free proteomics. Figure S4: relative ubiquitinated protein levels between Sh-LINC00578 and Sh-NC groups by coimmunoprecipitation.

Supplementary 2 Table S1: shRNA/siRNA sequence.

Supplementary 3 Table S2: PCR primers used in this study.

Supplementary 4 Table S3: antibody list.
