# Supplementary material for: Long Noncoding RNA LINC00578 Inhibits Ferroptosis in Pancreatic Cancer via Regulating SLC7A11 Ubiquitination
Source: Oxid Med Cell Longev. 2023 Feb 14;2023:1744102. doi: 10.1155/2023/1744102 (PMC9950792; doi:10.1155/2023/1744102)
Supplement: Supplementary 1 — Figure S1: Kaplan-Meier analysis of the correlation of LINC00578 expression with disease-free survival (DFS) (P = 0.002), from the cBioPortal (TCGA Firehose Legacy) cohort. Figure S2: relative expression levels of LINC00578 in PATU8988 cells with LINC00578 overexpression and PL45 cells with LINC00578 knockdown. Figure S3: differential expressed proteins between Sh-LINC00578 and Sh-NC groups selected by label-free proteomics. Figure S4: relative ubiquitinated protein levels between Sh-LINC00578 and Sh-NC groups by coimmunoprecipitation. [file 1744102.f1.pdf]

A

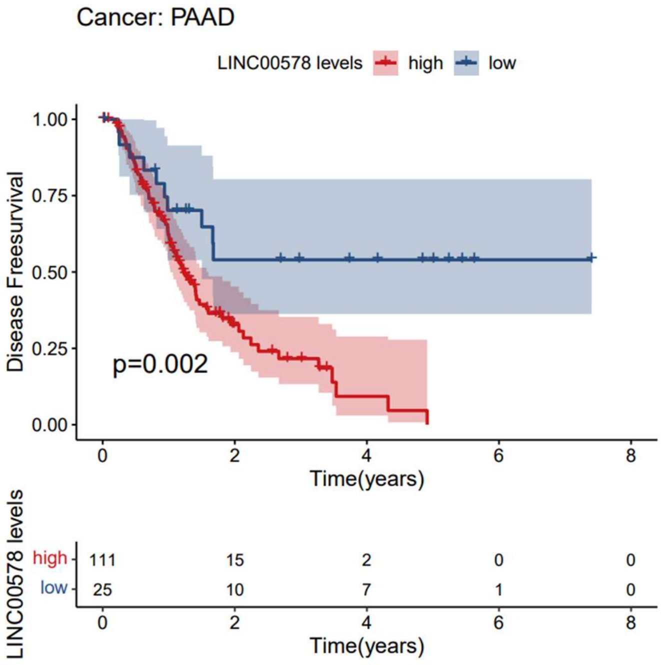

Figure S1. Kaplan-Meier analysis of the correlation of LINC00578 expression with disease-free survival (DFS) (P = 0.002) , from cBioportal (TCGA Firehose Legacy) cohort.

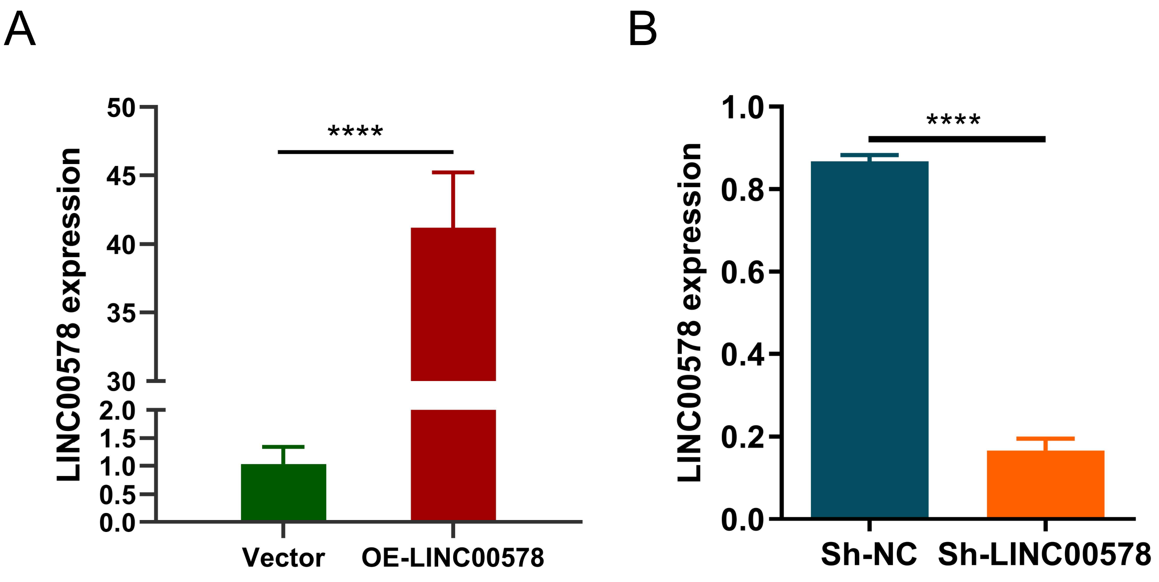

Figure S2. Relative expression levels of LINC00578 in PATU8988 cells with LINC00578 overexpression and PL45 cells with LINC00578 knockdown.

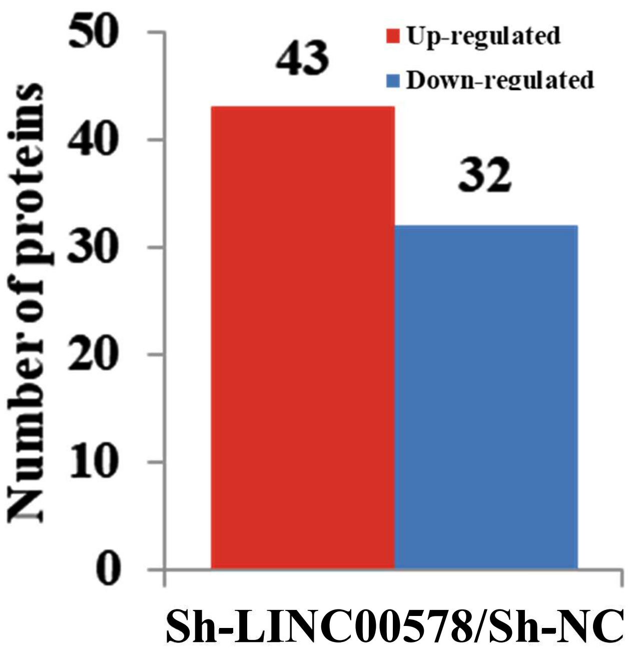

Down-regulated			Up-regulated		
Protein accession	Gene name	KD/NC Ratio	Protein accession	Gene name	KD/NC Ratio
Q8TDN6	BRIX1	0.637	Q9Y5Y2	NUBP2	1.571
O60925	PFDN1	0.629	Q13907	IDI1	1.513
P51571	SSR4	0.55	Q12907	LMAN2	1.656
Q5BJD5	TMEM41B	0.604	Q92925	SMARCD2	1.941
Q4G0N4	NADK2	0.614	Q13228	SELENBP1	1.717
P18077	RPL35A	0.562	Q7Z4H8	POGLUT3	1.594
P29317	EPHA2	0.567	P68363	TUBA1B	1.516
Q9Y295	DRG1	0.521	Q07960	ARHGAP1	1.594
P11717	IGF2R	0.552	Q9BW72	HIGD2A	2.145
Q9UH99	SUN2	0.524	Q14353	GAMT	1.519
P02794	FTH1	0.522	Q8N4Q1	CHCHD4	1.692
Q9BZK7	TBL1XR1	0.547	P51153	RAB13	2.268
O15031	PLXNB2	0.511	P18858	LIG1	1.782
Q9P1Y5	CAMSAP3	0.654	Q8TDP1	RNASEH2C	1.837
P62888	RPL30	0.497	P58107	EPPK1	2.003
P25440	BRD2	0.623	Q86Y39	NDUFA11	1.635
Q02750	MAP2K1	0.661	Q92817	EVPL	1.677
P42330	AKR1C3	0.646	Q8NFV4	ABHD11	1.821
P45880	VDAC2	0.553	Q7Z3T8	ZFYVE16	1.782
Q13347	EIF3I	0.577	P33993	MCM7	1.554
Q9Y277	VDAC3	0.574	P54105	CLNS1A	1.519
Q9H8H3	METTL7A	0.594	P04062	GBA	1.751
Q99873	PRMT1	0.618	Q9C005	DPY30	1.797
Q9Y3A5	SBDS	0.531	Q9H3G5	CPVL	1.809
P57076	CFAP298	0.407	P61024	CKS1B	1.509
P63172	DYNLT1	0.577	P84103	SRSF3	1.509
O43291	SPINT2	0.589	Q96I99	SUCLG2	2.086
Q7Z3J2	VPS35L	0.601	O95299	NDUFA10	1.608
P11166	SLC2A1	0.576	Q7Z5L9	IRF2BP2	1.618
P62873	GNB1	0.558	Q14257	RCN2	1.656
Q9UHG3	PCYOX1	0.66	P62081	RPS7	1.604
Q9BSH5	HDHD3	0.625	Q96K17	BTF3L4	1.681
			Q9NX40	OCIAD1	1.532
			Q5T6F2	UBAP2	1.594
			Q9NRZ9	HELLS	1.692
			P61970	NUTF2	1.509
			A3KMH1	VWA8	1.736
			P61960	UFM1	1.959
			Q14232	EIF2B1	1.558
			P43405	SYK	1.567
			Q9NZL4	HSPBP1	1.587
			Q9BWD1	ACAT2	1.509
			P08397	HMBS	2.058

Figure S3. Differential expressed proteins between Sh-LINC00578 and Sh-NC groups selected by Label-free proteomics

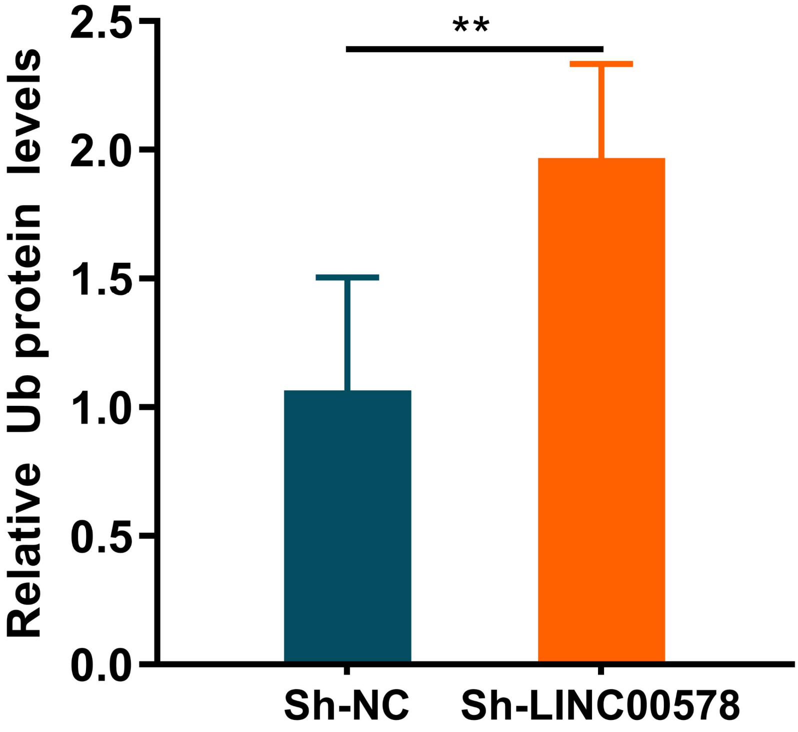

Figure S4. Relative ubiquitinated protein levels between Sh-LINC00578 and Sh-NC groups by coimmunoprecipitation
