# Supplementary material for: Long Noncoding RNA LINC00578 Inhibits Ferroptosis in Pancreatic Cancer via Regulating SLC7A11 Ubiquitination
Source: Oxid Med Cell Longev. 2023 Feb 14;2023:1744102. doi: 10.1155/2023/1744102 (PMC9950792; doi:10.1155/2023/1744102)
Supplement: Supplementary 2 — Table S1: shRNA/siRNA sequence. [file 1744102.f2.docx]

| Genes | sequences (5’-3’) |
| --- | --- |
| Sh-LINC00578  Sh-NC  Si-SLC7A11-sense | GCTGTCCACATTTCACTATCT  TTCTCCGAACGTGTCACGT  GGGAACAACUAUAAAGAAATT |
| Si-SLC7A11-antisense | UUUCUUUAUAGUUGUUCCCTT |
| Si-NC-sense | UUCUCCGAACGUGUCACGUTT |
| Si-NC-antisense | ACGUGACACGUUCGGAGAATT |

**Supplementary Table S1: ShRNA/SiRNA sequence**
