# Supplementary material for: Long Noncoding RNA LINC00578 Inhibits Ferroptosis in Pancreatic Cancer via Regulating SLC7A11 Ubiquitination
Source: Oxid Med Cell Longev. 2023 Feb 14;2023:1744102. doi: 10.1155/2023/1744102 (PMC9950792; doi:10.1155/2023/1744102)
Supplement: Supplementary 3 — Table S2: PCR primers used in this study. [file 1744102.f3.docx]

| **PCR Primers used in this study** | |
| --- | --- |
| Genes | Primer sequences (5’-3’) |
| LINC00578-F | TGTTAATAGGAGCCTGGTGAGTGGA |
| LINC00578-R | CCATTCCAGTGACAGGTTAGCACTC |
| U6-F | GCTTCGGCAGCACATATACTAAAAT |
| U6-R | CGCTTCACGAATTTGCGTGTCAT |
| GAPDH-F | AGAAGGCTGGGGCTCATTTG |
| GAPDH-R | AGGGGCCATCCACAGTCTTC |
| SLC7A11-F | CGGTGGTGTGTTTGCTGTCTC |
| SLC7A11-R | TGGTAGAGGAGTGTGCTTGCG |
| **Oligonucleotide sequence for RNA pulldown** | |
| LINC00578-  sense-F | **TAATACGACTCACTATAGGG**CGGTGTAGCACGATGGAATCTGGTGGCTTA |
| LINC00578-  sense-R | ATTTTTGAGACAGAGTCTCACTCTGTCGCCCAGG |
| LINC00578-  antisense-F | **TAATACGACTCACTATAGGG**ATTTTTGAGACA GAGTCTCACTCTGTC GCCCAGG |
| LINC00578-  antisense-R | CGGTGTAGCACGATGGAATCTGGTGGCTTA |

**Supplementary Table S2: PCR Primers used in this study**
