# Supplementary material for: Long Noncoding RNA LINC00578 Inhibits Ferroptosis in Pancreatic Cancer via Regulating SLC7A11 Ubiquitination
Source: Oxid Med Cell Longev. 2023 Feb 14;2023:1744102. doi: 10.1155/2023/1744102 (PMC9950792; doi:10.1155/2023/1744102)
Supplement: Supplementary 4 — Table S3: antibody list. [file 1744102.f4.docx]

**Supplementary Table S3: Antibody list**

| Primary antibodies | MW (kDa) | Dilution | Company/Catalog | Secondary Antibodies | Dilution |
| --- | --- | --- | --- | --- | --- |
| SLC7A11 | ≈55 | 1:1000 | Ptgcn/ 26864-1-AP | Goat Anti Rabbit IgG/HRP | 1:4000 |
| P53 | ≈53 | 1:1000 | Cst/ 9282 | Goat Anti Rabbit IgG/HRP | 1:4000 |
| C-MYC | ≈49 | 1:2000 | Ptgcn/ 10828-1-AP | Goat Anti Rabbit IgG/HRP | 1:4000 |
| GPX4 | ≈19 | 1:1000 | Ptgcn/ 14432-1-AP | Goat Anti Rabbit IgG/HRP | 1:4000 |
| Survivin | ≈16 | 1:1000 | Cst/ 2803 | Goat Anti Rabbit IgG/HRP | 1:4000 |
| GAPDH | ≈36 | 1:10000 | Ptgcn/ 60004-1-Ig | Goat Anti Mouse IgG/HRP | 1:4000 |
| Ubiquitin | ≈26 | 1:1000 | Ptgcn/ 10201-2-AP | Goat Anti Rabbit IgG/HRP | 1:4000 |
